# Expression of miR-17-92 enhances anti-tumor activity of T-cells transduced with the anti-EGFRvIII chimeric antigen receptor in mice bearing human GBM xenografts

**DOI:** 10.1186/2051-1426-1-21

**Published:** 2013-12-16

**Authors:** Masasuke Ohno, Takayuki Ohkuri, Akemi Kosaka, Kuniaki Tanahashi, Carl H June, Atsushi Natsume, Hideho Okada

**Affiliations:** 1 Brain Tumor Program, University of Pittsburgh Cancer Institute, 1.19E Research Pavilion at the Hillman Cancer Center, 5117 Centre Ave, Pittsburgh, PA 15213, USA; 2 Department of Neurosurgery, Nagoya University School of Medicine, 65 Tsurumai-cho, Showa-ku, Nagoya 466-8550, Japan; 3 Department of Pathology and Laboratory Medicine, Abramson Family Cancer Research Institute, University of Pennsylvania Perelman School of Medicine, Philadelphia, PA 19104, USA; 4 Department of Neurological Surgery, University of Pittsburgh School of Medicine, 200 Lothrop Street, Pittsburgh, PA 15213, USA; 5 Department of Surgery, University of Pittsburgh School of Medicine, 200 Lothrop Street, Pittsburgh, PA 15213, USA; 6 Department of Immunology, University of Pittsburgh School of Medicine, 200 Lothrop Street, Pittsburgh, PA 15213, USA

**Keywords:** microRNA, miR-17-92, Chimeric antigen receptor, Glioblastoma, Adoptive immunotherapy

## Abstract

**Background:**

Expression of miR-17-92 enhances T-cell survival and interferon (IFN)-γ production. We previously reported that miR-17-92 is down-regulated in T-cells derived from glioblastoma (GBM) patients. We hypothesized that transgene-derived co-expression of miR17-92 and chimeric antigen receptor (CAR) in T-cells would improve the efficacy of adoptive transfer therapy against GBM.

**Methods:**

We constructed novel lentiviral vectors for miR-17-92 (FG12-EF1a-miR-17/92) and a CAR consisting of an epidermal growth factor receptor variant III (EGFRvIII)-specific, single-chain variable fragment (scFv) coupled to the T-cell receptor CD3ζ chain signaling module and co-stimulatory motifs of CD137 (4-1BB) and CD28 in tandem (pELNS-3C10-CAR). Human T-cells were transduced with these lentiviral vectors, and their anti-tumor effects were evaluated both *in vitro* and *in vivo*.

**Results:**

CAR-transduced T-cells (CAR-T-cells) exhibited potent, antigen-specific, cytotoxic activity against U87 GBM cells that stably express EGFRvIII (U87-EGFRvIII) and, when co-transduced with miR-17-92, exhibited improved survival in the presence of temozolomide (TMZ) compared with CAR-T-cells without miR-17-92 co-transduction. In mice bearing intracranial U87-EGFRvIII xenografts, CAR-T-cells with or without transgene-derived miR-17-92 expression demonstrated similar levels of therapeutic effect without demonstrating any uncontrolled growth of CAR-T-cells. However, when these mice were re-challenged with U87-EGFRvIII cells in their brains, mice receiving co-transduced CAR-T-cells exhibited improved protection compared with mice treated with CAR-T-cells without miR-17-92 co-transduction.

**Conclusion:**

These results warrant the development of novel CAR-T-cell strategies that incorporate miR-17-92 to improve therapeutic potency, especially in patients with GBM.

## Background

Although the central nervous system (CNS) is often considered to be immunologically privileged [1], recent vaccine studies in patients with malignant glioma reviewed in [2,3], including ours [4], have demonstrated promising results. However, vaccine efficacy, which relies on intact host-immune activity, can suffer from systemic suppression of immunity due to tumor expression of immunosuppressive cytokines as well as chemo- and radiotherapy. On the other hand, adoptive cell transfer (ACT) therapy with autologous T-cells, especially using T-cells transduced with chimeric antigen receptors (CARs), has shown promise in pilot hematologic cancer trials [5-8]. ACT with CAR-T-cells may be particularly suitable for patients with glioblastoma (GBM) because the specificity, number, and functional phenotype of cells prepared *ex vivo* can be controlled and manipulated far better than native T-cells induced by *in vivo* immunization.

CARs offer several advantages compared with traditional T-cell receptor (TCR)-mediated targeting of tumor antigens. Unlike TCRs, CARs do not require antigen-presentation by major histocompatibility complex (MHC), which is often down-regulated in gliomas [9,10]. CARs have evolved over the last decade, with progressively increasing co-stimulatory activity. In addition to a single signaling unit derived from the CD3ζ chain, second generation CARs incorporate the intracellular domain of a co-stimulatory molecule, CD28 or tumor necrosis factor (TNF) receptor family member, CD137 (4-1BB). Subsequent incorporation of both CD28 and CD137 has enhanced the ability of these receptors to stimulate cytokine secretion and hence the antitumor efficacy of third generation CARs [11-14]. Recently, autologous CAR-modified T-cells re-infused into 3 patients with refractory chronic lymphocytic leukemia led to complete remission in 2 of the 3 patients [5,6]; engineered cells persisted at high levels for 6 months and continued to express the CAR [6]. These data provide strong justification for the pursuit of CAR-based therapy for other types of cancer, such as GBM.

To maximize the safety of the CAR approach, it is critical to select target antigens that allow specific targeting of tumor cells [15]. Epidermal growth factor receptor variant III (EGFRvIII) results from the in-frame deletion of exons 2–7 and the generation of a novel glycine residue at the junction of exons 1 and 8 within the extra-cellular domain of the EGFR, thereby creating a tumor-specific and immunogenic epitope reviewed in [16,17]. EGFRvIII expression has been seen in many tumor types, including GBM, but is rarely observed in normal tissue. EGFRvIII is expressed in 24% to 67% of GBM cases, and in patients surviving ≥1 year, the expression of EGFRvIII is an independent negative prognostic indicator [18,19].

With regard to the suitable effector T-cell phenotype for anti-CNS tumor immunotherapy, our previous studies have demonstrated that expression of IFN-γ by T-cells [20] and the resulting expression of IFN-inducible protein (IP)-10, also known as CXCL10, in the CNS tumor environment [21,22], are critically important. These studies led us to investigate novel microRNA (miR)-mediated mechanisms in the tumor-microenvironment [23] and in T-cell-based immunotherapy for GBM, as miRs can mediate a variety of biological responses, including immune responses [24]. We have demonstrated that miR-17-92, which is known to promote cell survival and proliferation [25], is the most significantly up-regulated microRNA in IFN-γ-producing T helper type-1 (Th1) cells but is down-regulated in patients with GBM [26]. Our data also demonstrated that transduction of human T-cells to express miR-17-92 at high levels promotes their survival and production of IFN-γ [26].

Based on these data, we evaluated our hypothesis that transgene-derived overexpression of miR-17-92 improves the therapeutic efficacy of CAR-transduced T-cells in a preclinical model of GBM.

## Results

### Construction of lentiviral vectors for EGFRvIII-specific CAR and miR-17-92

**Figure 1:**
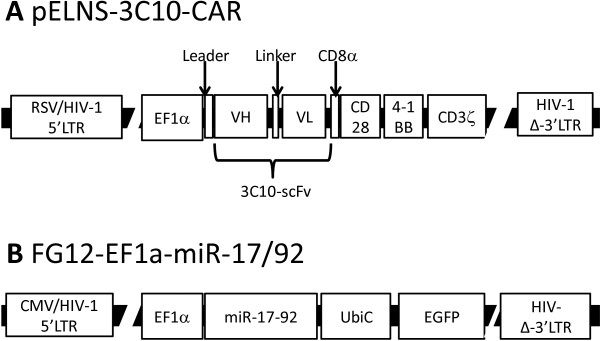
**Schematic diagrams of lentiviral vectors for 3C10-CAR and miR-17-92. (A)** The 3C10-CAR-expressing vector pELNS-3C10-CAR. The EF1α promoter drives the CAR construct containing the 3C10 scFv for targeting of EGFRvIII. The CAR also incorporates CD28 as well as 4-1BB and CD3ζ domains. **(B)** The miR-17-92-expressing lentiviral vector FG12-EF1a-miR-17/92. miR-17-92 is expressed under the control of the EF1a promoter. The vector also contains the human UbiC promoter driving the EGFP as a marker gene for transduced cells. Abbreviations: RSV/HIV-1 5’LTR = Hybrid RSV promoter-R/U5 long terminal repeat; EF1α = Human elongation factor 1α-subunit promoter; VH = Variable region in the heavy chain of the 3C10 immunoglobulin; VL = Variable region in the light chain of the 3C10 immunoglobulin; HIV-1 Δ-3′LTR = Self-inactivating 3′ long terminal repeat with deletion in U3 region; CMV/HIV-1 5′LTR = Hybrid CMV promoter-R/U5 long terminal repeat; UbiC = Ubiquitin C promoter.

We generated a lentiviral vector for a CAR that recognizes the EGFRvIII through a single-chain variable fragment (scFv) derived from human EGFRvIII-specific monoclonal antibody (mAb) 3C10 (pELNS-3C10-CAR; Figure 1A). In this construct, the EF1α promoter drives the CAR fusion protein integrating the 3C10-derived scFV, CD28 trans-membrane (TM), and intracellular domains (ICD) as well as the 4-1BB ICD and CD3ζ domains. We also created a lentiviral miR-17-92 construct using the FG12-based self-inactivating (SIN) vector (FG12-EF1a-miR-17/92; Figure 1B). In this vector, the EF1α promoter drives miR-17-92, and the human UbiC promoter drives enhanced green fluorescence protein (EGFP) marker gene to track transduced cells.

### *In vitro* characterization of human T-cells transduced with the CAR and miR-17-92

**Figure 2:**
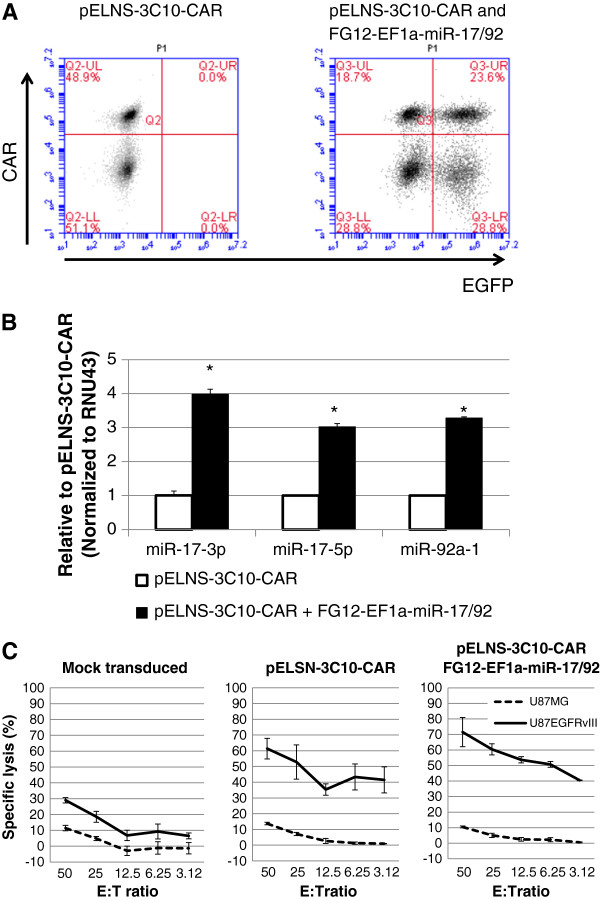
**Functional expression of lentivirally transduced 3C10-CAR and miR-17-92 in human T cells.** CD3^+^ T cells were transduced with pELNS-3C10-CAR alone or with both pELNS-3C10-CAR and FG12-EF1a-miR-17/92 as described in Methods. **(A)** Transduced T-cells were evaluated by flow cytometry for expression of 3C10-CAR and miR-17-92 by anti-mouse (Fab’)2 antibody and EGFP, respectively. **(B)** Expression levels of the miR-17-92 cluster members miR-17-3p, miR-17-5p, and miR-92a-1 in transduced T cells were measured by qRT-PCR. Mean ± SD values of 3 replicate measurements from one of three experiments with similar results are depicted. * indicates p < 0.05 between the two groups using student t-test. **(C)** EGFRvIII-specific cytotoxic activities of transduced T cells evaluated by a 12-h ^51^Cr-release assay at various E:T ratios against ^51^Cr-labeled U87-EGFRvIII or control U87 cells. Control cells were Mock (EGFP)-transduced T-cells. Values indicate mean ± SD in triplicated wells.

We transduced healthy donor-derived CD3^+^ T-cells with pELNS-3C10-CAR and evaluated expression levels of the transgene (Figure 2A, Left). Using anti-mouse F(ab’)2 Ab, which is specific for the 3C10-derived scFv on human T-cells, we detected nearly half (48.9%) of the T-cells expressing the 3C10-derived scFv on their surface.

To obtain human T-cells expressing both the CAR and transgene-derived miR-17-92, we co-transduced CD3^+^ T-cells with pELNS-3C10-CAR and FG12-EF1a-miR-17/92 by sequential infection of the two lentiviral vectors. At 24 hours after the initial transduction with pELNS-3C10-CAR, we transduced the T-cells with FG12-EF1a-miR-17-92. We observed that approximately one quarter (23.6%) of the total T-cells expressed both CAR and EGFP (hence the transgene-derived miR-17-92; Figure 2A, Right). For subsequent *in vitro* studies, we enriched CAR-transduced T-cells (CAR-T-cells) by biotinylated anti-mouse F(ab’)2 Ab and anti-biotin MACS. By real-time PCR, we also detected 3–4 fold higher expression of miR-17-92 in the miR-17-92-co-transduced CAR-T-cells compared with T-cells transduced with the CAR alone (Figure 2B).

While the mock-transduced T-cells showed only background levels of lysis against both parental U87 (EGFRvIII-negative) and U87-EGFRvIII cells, T-cells transduced with the CAR demonstrated potent and specific lysis of EGFRvIII-expressing U87 human GBM cells (U87-EGFRvIII), with only background levels of cytotoxic effects against parental U87 cells (Figure 2C). Co-transduction of CAR-T-cells with miR-17-92 did not significantly enhance their specific cytotoxic activity against U87-EGFRvIII target cells.

### miR-17-92 co-transduction enhances IFN-γ release and reduces inhibition of proliferation of CAR-T-cells in the presence of TMZ

In our previous study [26], CD4^+^ T-cells derived from miR-17-92 transgenic mice demonstrated increased IFN-γ production when compared with counterparts derived from wild type mice, and transfection of human Jurkat T-cells with miR-17-92 led to enhanced resistance to activation-induced cell death. To extend these observations in a more clinically relevant setting, we evaluated whether co-transduction of primary human CAR-T-cells with miR-17-92 confers improved IFN-γ production and cell proliferation. In addition, we determined the effects on apoptosis after exposure to TMZ, a chemotherapy agent that is part of the standard-of-care in the treatment of GBM [27].

**Figure 3:**
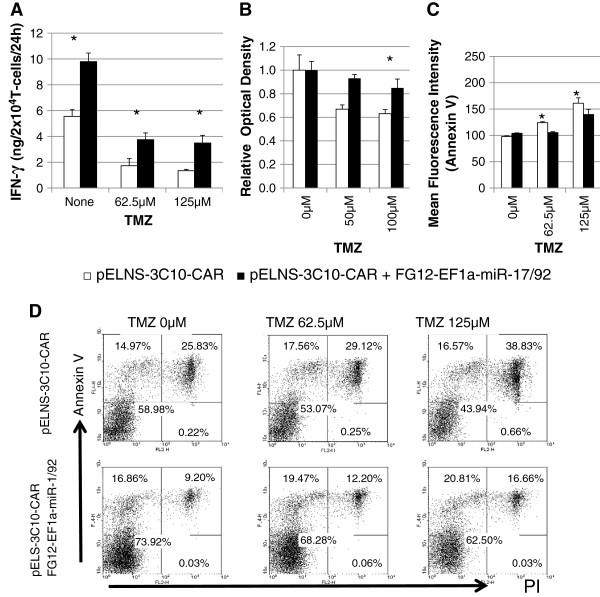
**Co-expression of miR17-92 in CAR-T-cells confers resistance to suppressive effects of TMZ.** CAR-T-cells and those co-transduced with miR-17-92 were co-cultured with aAPCs expressing EGFRvIII in the presence of the indicated concentrations of TMZ. **(A-C)** Open bars and closed bars represent results from CAR-T-cells (without miR-17-72) and miR-17-92 co-transduced CAR-T-cells, respectively. **(A)** IFN-γ produced by the transduced T cells during the last 24 h of 96 h co-culture. * indicates P < 0.05 for comparison of the two cell types at the given condition. TMZ significantly suppressed IFN-γ production in each of the two cell types (P < 0.05 by one-way ANOVA). **(B)** Relative proliferation levels between the groups were evaluated by WST1 assay following the 3-day co-culture. **(C and D)** Apoptotic death of CAR-T-cells evaluated by Annexin-V and PI. **(C)** Mean fluorescent intensity for Annexin-V on CAR-T-cells exposed to TMZ. Values indicate mean ± SD in triplicate wells. (* indicates P < 0.05) **(D)** Flow-cytometric histograms for Annexin-V^+^ and/or PI^+^ in one of the three experiments (all had similar results).

Upon EGFRvIII-specific stimulation without TMZ, the co-transduced CAR-T-cells expressed higher levels of IFN-γ compared with CAR-T-cells without miR-17-92 co-transduction (Figure 3A). When the cells were exposed to escalating doses of TMZ, both cell types demonstrated decreased IFN-γ production levels. However, the co-transduced CAR-T-cells consistently demonstrated higher IFN-γ production levels compared with CAR-T-cells without miR-17-92 co-transduction at each of the evaluated dose levels.

We next evaluated the effects of miR-17-92 co-transduction on the proliferation of CAR-T-cells in the presence of TMZ in culture (Figure 3B). To specifically evaluate the impact of TMZ on the CAR-T-cell proliferation, we depicted the proliferation rate of the cells in each group relative to the proliferation of the same cells without TMZ. With increasing concentrations of TMZ, proliferation of miR-17-92 co-transduced CAR-T-cells was significantly less inhibited compared with the control CAR-T-cells. Without TMZ, miR-17-92-co-transduced CAR-T-cells demonstrated a trend toward a more rapid rate of proliferation compared with control CAR-T-cells, but the difference was not significant (data not shown).

We also evaluated whether miR-17-92-co-transduction would render CAR-T-cells more resistant to TMZ-induced apoptosis. To this end, we conducted flow-cytometric assessments of Annexin V^+^ and propidium iodide (PI) staining of CAR-T-cells in the presence of increasing concentrations of TMZ (Figure 3C and D). We observed a dose-dependent increase of both early (Annexin V^+^PI^-^) and late (Annexin V^+^PI^+^) apoptotic cells; miR-17-92-co-transduced CAR-T-cells demonstrated a lesser degree of apoptotic changes compared with control CAR-T-cells.

### Intravenous injection of CAR-T-cells in combination with TMZ leads to complete remission of established U87-EGFRvIII tumors in NSG mice

**Figure 4:**
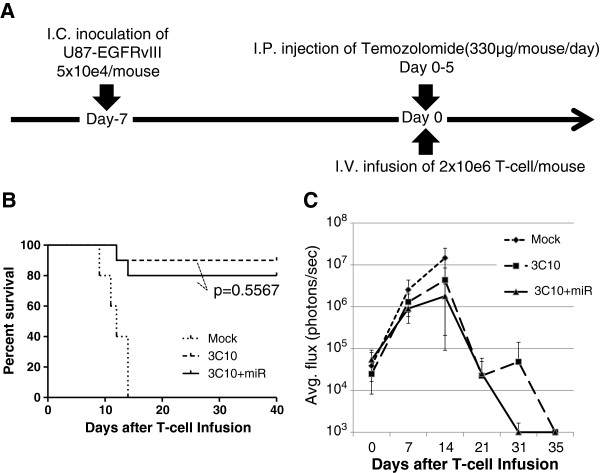
**Robust therapeutic effects of CAR-T-cells in mice bearing U87-EGFRvIII tumors. (A)** Schematic of the experimental protocol. NSG mice received i.c. inoculation of 5×10^4^ U87-EGFRvIII-Luc cells on day -7 and subsequently received a single i.v. infusion of 2×10^6^ T cells transduced with pELNS-3C10-CAR alone or with both pELNS-3C10-CAR and FG12-EF1a-miR-17/92 or mock vector on day 0. All mice received i.p. administration of TMZ (0.33 mg/mouse/day) on days 0–4. **(B)** Kaplan-Meier analysis. Median survival of the mice treated with CAR-T cells (with or without co-transduction of miR-17-92; n = 10/group) was significantly greater compared with the mice treated with mock-transduced T cells (p < 0.05). All mock-transduced mice (n = 5) died by day 21. **(C)** Longitudinal measurements of tumor-derived mean photon flux ± SD. The background luminescence level (up to 10^3^ p/s) was defined based on the levels observed in non-tumor-bearing mice imaged in parallel with the tumor-bearing mice. Results are from one of two independent experiments with similar results.

We next evaluated the efficacy of CAR-T-cells in immunocompromised NOD/scid/γc(-/-) (NSG) mice bearing established (day 7) intracranial U87-EGFRvIII tumors. Mice received a single intravenous (i.v.) infusion of miR-17-92 co-transduced CAR-T-cells, CAR-T-cells without co-transduction of miR-17-92, or mock-transduced T-cells (2 × 10^6^/mouse) via the tail vein. As newly diagnosed GBM patients routinely receive TMZ therapy, we also administered intraperitoneal (i.p.) daily injections of TMZ for 5 days starting on the day of T-cell infusion (Figure 4A and Additional file 1: Figure S1).

TMZ treatment itself was ineffective: all the control mice receiving TMZ and mock-transduced T-cells died within 3 weeks after the T-cell infusion (Figure 4B). Although 1 of 10 mice with CAR-T-cells and 2 of 10 mice with miR-17-92 co-transduced CAR-T-cells died by day 22 as a result of tumor progression, all the other mice in these groups survived longer than 40 days. Indeed, bioluminescence imaging (BLI) signal levels in these surviving mice diminished to background levels by day 35, consistent with tumor eradication in these mice (Figure 4C). There was not a statistically significant difference in survival of the mice receiving miR-17-92-co-transduced CAR-T-cells versus CAR-T-cells without miR-17-92 co-transduction (log-rank test: p = 0.5567).

We also evaluated the effect of 3C10-CAR-T-cells without TMZ treatment (Additional file 1: Figure S2). While direct intra-tumoral injection of 3C10-CAR-T-cells eradicated the U87-EGFRvIII tumors without TMZ treatment (data not shown), i.v. infusion of 3C10-CAR-T-cells without TMZ was ineffective, regardless of whether the T-cells were co-transduced with miR-17-92, suggesting U87-EGFRvIII tumors were extremely aggressive without TMZ-treatment (Additional file 1: Figure S2).

### miR-17-92 co-transduced CAR-T-cells confers long-term protection against U87-EGFRvIII tumors in mice

**Figure 5:**
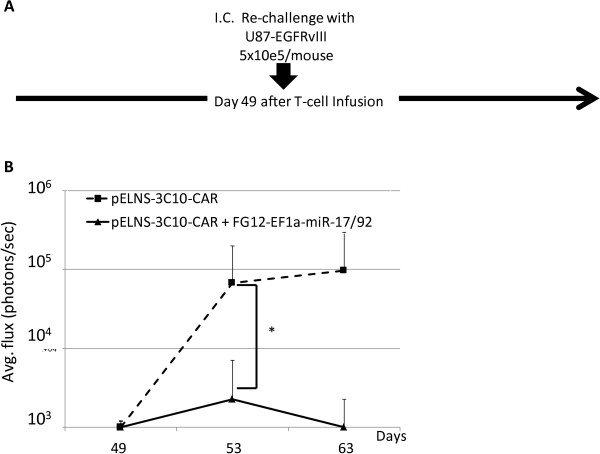
**Ectopic expression miR-17-92 in CAR-T cells confers improved protection following tumor re-challenge. (A)** Schematic of the experimental protocol. CAR-T-cell-treated mice that survived at least for 35 days in the experiment shown in Figure 4 [4 mice receiving 3C10-T-cells without miR and 3 mice treated with T-cells co-transduced with 3C10 plus miR-17-92] received i.c. re-challenge with U87-EGFRvIII-Luc cells (5×10^5^/mice) on day 49. No additional CAR T-cells were injected. **(B)** Longitudinal measurements of tumor-derived mean photon flux ± SD from the 2 groups of mice. The background luminescence level (up to 10^3^ p/s) was defined based on the levels observed in non-tumor-bearing mice imaged in parallel with tumor-bearing mice in treatment groups. *p < 0.05 by Wilcoxon rank-sum test.

To determine whether CAR-T-cells can provide long-term protection against U87-EGFRvIII tumors, we re-challenged the survivors in the experiment described above (Figure 4) with inoculation of U87-EGFRvIII cells in the contra-lateral hemisphere of the brain on day 49 post T-cell infusion (Figure 5). Prior to this experiment, our preliminary data demonstrated that i.v. infused 3C10-CAR-T-cells efficiently migrated to intracranial U87-EGFRvIII tumor tissue (Additional file 1: Figure S3) and persisted, with transgene-derived expression of the CAR in the spleen for more than 60 days (Additional file 1: Figure S4). While the re-challenged tumor cells grew in all four mice treated with CAR-T-cells, none of three mice treated with miR-17-92-co-transduced CAR-T-cells demonstrated BLI signal beyond background levels. These results strongly suggest that co-transduction of miR-17-92 cluster promotes the long-term persistence and function of CAR-T-cells, thereby providing prolonged protection against tumor growth.

## Discussion

To our knowledge, this is the first gene transfer study evaluating the potential of miR-17-92 to enhance cancer immunotherapy. Our data show that co-expression of miR-17-92 confers improved resistance to the effects of TMZ on T-cells. *In vivo*, T-cells co-transduced with both 3C10-CAR and miR-17-92 demonstrated more persistent therapeutic effects compared with T-cells transduced with 3C10-CAR alone.

Lentiviral transduction of miR-17-92 in our study confers ectopic over-expression of the miR-cluster in transduced T-cells. During the physiological T-cell activation and differentiation processes, however, expression levels of endogenous miR-17-92 in T-cells appear to be tightly regulated. In human CD8^+^ T cells, miR-17-92 expression is high in naïve cells but diminishes as the cells differentiate [28]. In a mouse model of lymphocytic choriomeningitis virus infection, miR-17-92 is strongly up-regulated following T-cell activation but is later down-regulated following clonal expansion and is further silenced during memory development [29]; in this study, miR-17-92 was necessary for rapid T-cell expansion and IFN-γ expression, and failure to down-regulate miR-17-92 led to a gradual loss of memory cells and defective central memory cell development [29]. These observations are not necessarily consistent with our results, as we observed the persistence of miR-17-92-co-transduced CAR-T-cells and protection of the mouse brain against tumorigenesis when re-challenged with U87-EGFRvIII cells. It is possible that our observation is attributable to the combined effects of miR-17-92 and the co-stimulatory molecules provided by the CAR.

We did not evaluate the specific function of each miR in the miR-17-92 cluster. However, it has been recently shown that miR-17 and miR-19b are critical for Th1 responses, with miR-17 and miR-19b targeting TGFβRII and Pten (phosphatase and tensin homolog), respectively [30,31]. These two miRs mediate T-cell proliferation, protection from activation-induced cell death, IFN-γ production, and inhibition of regulatory T-cell development [30,31]. Hence, although we were unable to detect down-regulation of TGFβRII in miR-17-92-transduced T-cells (data not shown), we must consider whether miR-17 and miR-19b alone are sufficient to mediate the observed enhancement of anti-GBM efficacy in our CAR therapy. Furthermore, glucocorticoids, which are often used in the management of GBM, repress the expression of miR-17-92 in lymphocytes, thereby up-regulating proapoptotic Bim as a mechanism inducing apoptotic death of lymphoid cells [32]. Together with the results from our current study, these data provide further rationale for the incorporation of miR-17-92 CAR designs.

Although miR-17-92 has been described as an oncogenic miR [33], miR-17-92 overexpression itself is known not to be oncogenic in lymphocytes [25]. Indeed, we did not observe any uncontrolled proliferation of miR-17-92-transduced T-cells in the current study. Nonetheless, as an alternative approach for improved safety, transient transduction of T-cells with miR-17-92 itself, instead of lentiviral stable transfer, and multiple injections of these T-cells, may represent a reasonable approach without the associated safety concerns of integrating viral vectors [34].

With regard to the inclusion of co-stimulatory signals in CAR constructs, the current study focused on the use of a “third generation” system. It would be interesting to directly compare second generation (i.e., the CAR construct with 4-1BB plus CD3zeta chain without CD28) versus third generation CARs in our tumor model, and we have in fact undertaken such a comparative study (manuscript in preparation).

In support of EGFRvIII-targeted CARs for therapy of GBM, Morgan *et al.* recently evaluated scFv sequences derived from seven different anti-EGFRvIII mAbs, including 3C10 and humanized 139, in γ-retroviral CARs [35]. Based on their *in vitro* characterization, the 3C10 and the 139 CARs were two of the three clones that yielded specific IFN-γ production in response to EGFRvIII-expressing target cells but not to cells expressing wild-type *EGFR*. However, they did not evaluate the efficacy of EGFRvIII CAR-T cells in animal models.

Of course, EGFRvIII is expressed only in a subpopulation of GBM patients and in only a fraction of GBM cells, even in “EGFRvIII-positive” cases [19]. Although a recent report on a phase II vaccine study targeting the EGFRvIII epitope in newly diagnosed GBM patients demonstrated encouraging results as reflected in overall survival [36], immunotherapy targeting EGFRvIII as a single target will likely result in growth of GBM cells that have down-regulated the immunotherapy-targeted antigen [36]. Hence, we are currently working to develop CARs that target other GBM-relevant antigens. Several previous studies have developed CARs against GBM-associated antigens, such as IL-13Rα2 [37,38], HER-2 [39], and EphA2 [40]. We anticipate that an effective CAR therapy would use T-cells capable of resisting GBM-induced suppression mechanisms and targeting multiple antigens, such that the infused T-cells would exhibit effective and sustained therapeutic effect against tumors with heterogeneous antigen-expression profiles.

Although EGFRvIII is an attractive target for GBM therapy, a GBM-derived cell line that preserves EGFRvIII expression without transgene-derived EGFRvIII had not existed until Schulte *et al.* recently established such a cell line without EGF supplementation in the culture [41]. Additional studies are warranted to evaluate the potency of CARs that target EGFRvIII using GBM cells that express endogenous EGFRvIII, such as that conducted by Morgan *et al.*[35].

To evaluate the effects of EGFRvIII stimulation on CAR-T-cells, we used an artificial antigen-presenting cell (aAPC) line K562-CD32-CD80-41BBL [42] that had been transduced with EGFRvIII. aAPC offers a reproducible, cost-effective, and convenient method for polyclonal and antigen-specific expansion of human T cells for adoptive immunotherapy. Although the possibility exists that aAPCs could introduce confounding signals, we were able to obtain similar results when we used CD28/CD3 beads for T-cell stimulation (data not shown), suggesting no such signals from the aAPCs disturbed our data.

Our *in vivo* data demonstrate that the therapeutic efficacy of CAR-T-cells can be achieved only with pretreatment with TMZ. We speculate that the major effect of TMZ therapy is a reduction in tumor burden, but TMZ could enhance the efficacy of CAR-T-cell therapy through other mechanisms. As contrasting results have been reported with regard to the role of TMZ in glioma immunotherapy [43,44], we are currently evaluating the effects of TMZ specifically on the glioma microenvironment using syngeneic glioma models. Human GBM patients receive oral administration of 150–200 mg/m^2^/day as standard-of care [27], in which case peak serum TMZ concentrations of up to 100 μM [45]. We used these clinically relevant concentrations of TMZ in designing our *in vitro* studies to evaluate the effects of TMZ.

Other experimental parameters must be taken into account to optimize i.v. infused T-cell therapy in gliomas, such as factors that promote the homing of T-cells to the tumor (e.g., T-cell expression of very late activation antigen (VLA)-4 [46,47] and chemokine receptor CXCR3 [21,22]). While these factors cannot be precisely evaluated in our NSG mouse model, we will consider these markers in optimizing clinical trials for glioma CAR therapy.

## Conclusions

Our 3^rd^ generation CAR targeting EGFRvIII demonstrated an antigen-specific and potent efficacy in mice bearing EGFRvIII-expressing GBM xenografts. Furthermore, co-expression of miR-17-92 in CAR-T-cells enhanced durability of the therapeutic response. Our study supports further evaluation of CAR therapy that integrates miR-17-92, with a focus on the safe and effective delivery of the miR-17-92 cluster in CAR-transduced T-cells.

## Methods

### Cell lines

The GBM cell line U87-EGFRvIII was kindly provided by Dr. Shiyuan Chang (Northwestern University, Chicago, IL). The aAPC line K562-CD32-CD80-41BBL [42] and U87MG cell lines were maintained in RPMI-1640 medium containing 10% fetal bovine serum (FBS) and penicillin/streptomycin. Geneticin was added to maintain the U87-EGFRvIII cell line. The U87-EGFRvIII-Luc cell line was generated by transduction of U87-EGFRvIII cells with a recombinant lentivirus encoding *luciferase* of Photinuspyralis (provided by Dr. Stephen H. Thorne at University of Pittsburgh, Pittsburgh, PA) and subsequent selection by blasticidin for a clone expressing the highest level of luciferase.

### Construction of SIN lentiviral vectors

The mAb 3C10 was originally developed by the immunization of mice with a 14-amino acid peptide that incorporates the EGFRvIII-specific fusion junction [48]. Subsequently, its scFv was cloned [49]. The scFv portion in the pELNS-SS1CD28r1BBZeta [12] was replaced with the cDNA for the 3C10 scFV by gene synthesis (Genscript, Piscataway, NJ) to create pELNS-3C10-CAR. FG12-EF1α-miR-17/92 was generated based on FG12 (obtained from Chang-Zhen Chen at Stanford University through Addgene, plasmid #11375). PCR-amplified EF1α promoter fragment (with XbaI site on 5′ end and XhoI site on 3′ end) and the miR-17-92 pre-miRNA (synthesized by GenScript Corporation, Piscataway, NJ; with XhoI site on 5′ end and PacI site on 3′ end) were subcloned into the FG12 cassette using its XbaI and PacI sites.

### Preparation of lentiviral vectors

HEK293T cells (8 × 10^6^) were plated on 175 cm^2^ flask. At 24 h, SIN vector, pMDLg/pRRE, pRSV-Rev, and pMD2.G were co-transfected by X-tremeGENE 9 (Roche Applied Science Roche, Penzberg, Germany). Supernatant was collected at 48 h, mixed with PEG-it™ Virus Precipitation Solution (5×) (SBI, San Jose, CA), and incubated for 24 h at 4°C. The supernatant/PEG-it mixture was then centrifuged at 1500 × g for 30 minutes at 4°C, and the pellet was re-suspended in 1/10 of the original volume using cold, sterile medium containing 25 mM HEPES buffer at 4°C and stored at -80°C.

### T-cell isolation

Human T-cells were isolated from healthy donor-derived peripheral blood mononuclear cells by negative selection using the Pan T-Cell Isolation Kit II (Miltenyi BiotecInc., Auburn, CA).

### Transduction of T-cells by lentiviral vectors

The isolated T-cells (4×10^6^) were re-suspended in 4 ml medium per well of a 6-well plate and stimulated with Dynabeads Human T-Activator CD3/CD28 and IL-2 (Peprotech, 100 unit/ml) for 24 h. Next, the media (3 ml/well) was gently removed without disturbing clustering T cells, and the lentiviral pELNS-3C10-CAR vector supernatant (x10; 3 ml/well) was added (the final MOI ~ 5–6). T-cells were then cultured with the lentiviral vector for 24 h in RPMI1640 containing 10% FBS and IL-2 (100 unit/ml). For co-transduction, after the first transduction with the lentiviral CAR vector, the T-cells were subsequently infected with FG12-EF1α-miR-17/92 in RPMI1640 containing 10% FBS and IL-2 (100 unit/ml) for additional 24 h.

### Purification of 3C10-CAR-T-cells

CAR-T-cells were detected by biotin-SP-AffiniPure F(ab')2 fragment-specific goat anti-mouse IgG (Jackson Immuno Research Laboratories, West Grove, PA) and streptavidin-Phycoerythrin (PE) (BD Pharmingen, San Diego, CA). CAR-T-cells were enriched using the biotin-SP-AffiniPureF(ab')2 fragment-specific goat anti-mouse IgG and Streptavidin Micro Beads (Miltenyi Biotec Inc.) according to the manufacturer’s instructions. The purity of the isolated CAR-T-cells was > 90% (Additional file 1: Figure S5).

### Cell proliferation and cytokine release assays

aAPCs expressing EGFRvIII were generated by transfection of EGFRvIII-expression vector (PT3.5/CMV-EGFRvIII) using Amaxa Nucleofector System (Lonza, Walkersville, MD). Aliquots of 2 × 10^4^ T-cells were co-cultured with 2 × 10^4^ γ-irradiated aAPC in each well of 96-well plates and treated with graded concentrations of TMZ (Sigma-Aldrich, St. Louis, MO) for 72 h. Spectrophotometric quantification of T-cell proliferation was performed by using the metabolic proliferation reagent WST-1 (Roche Applied Science) according to the manufacturer’s instructions. The absorbance of the samples against a background control as blank (media) was measured at 450 nm by using a Multiscan Ascent microplate (ELISA) reader (Thermo Scientific, Rockford, IL). For evaluation of IFN-γ-release, following the 72 h co-culture, T-cell were cultured with new aAPC for an additional 24 h, and culture supernatants were assessed for IFN-γ using a specific ELISA kit (GE Healthcare Bio-Sciences).

### Target cell lysis

The susceptibility of U87MG and U87-EGFRvIII cells to cytolysis by the CAR-T-cells was evaluated using a standard 12-h ^51^Cr-release assay at various effector:target (E:T) ratios. The percentage of specific lysis was calculated as follows:

100 × (experimental release - spontaneous release)/

(maximum release - spontaneous release)

### Detection of apoptotic cells

Apoptotic cells were quantified by staining for Annexin V and propidium iodide (PI) using the Annexin V-APC apoptosis detection kit, according to the manufacturer’s instructions (BD Biosciences, San Jose, CA). The cells were analyzed with Accuri C6 (BD Accuri Cytometers, Ann Arbor, MI) or FACS Caliber (Becton Dickinson, Pont de Claix, France) flow cytometer.

### Therapy of mice bearing intracranial glioma xenografts

5- to 6-week-old NOD/Shi-scid,IL-2Rr KO Jic (NOG) female mice (Jackson Laboratory, Bar Harbor, ME) were used in the experiments. Animals were handled in the Animal Facility at the University of Pittsburgh per an Institutional Animal Care and Use Committee-approved protocol. The procedure has been described previously by us^21^. Briefly, using a stereotactic apparatus, mice received 5×10^4^ U87EGFRvIII cells/mouse in 2 μl PBS at 2 mm lateral to the bregma and 4 mm below the surface of the skull. Seven days after tumor inoculation, each mouse received i.v. infusion of 2 × 10^6^ CAR-T-cells, with or without co-transduction of miR-17-92, or mock-transduced T-cells via the tail vein. On days 0–4 after T-cell infusion, mice received i.p. administration of TMZ (333 μg/dose). Symptom-free survival following the tumor inoculation was monitored. The growth of U87EGFRvIII-Luc tumors in the brain was non-invasively monitored by BLI using the *in vivo* imaging system IVIS (PerkinElmer, Alameda, CA). Mice received i.p. injection of with 200 μl (15 mg/ml) of freshly thawed aqueous solution of D-Luciferin potassium salt (PerkinElmer), were anesthetized with isoflurane, and imaged for bioluminescence for 1 sec exposure time. Optical images were analyzed by IVIS living image software package. The mice that rejected the established U87-EGFRvIII tumors received intracranial (i.c.) re-challenge with 5×10^5^ U87-EGFRvIII cells at the same stereotactic coordinates on day 49.

### Real time-PCR

Total RNA was extracted using the Qiagen RNeasy kit (Qiagen, Hilden, Germany), and purity was confirmed with an A260/A280 ratio greater than 1.85. RNA was subjected to RT-PCR analysis using the TaqMan microRNA Reverse Transcription Kit, microRNA Assays (Applied Biosystems), and the Step One Real-Time PCR System (Applied Biosystems, Foster City, CA). The small nucleolar RNU43 was used as the housekeeping small RNA reference gene for human samples. All reactions were done in triplicate, and relative expression of RNAs was calculated using the ΔΔCT method.

### Statistical analyses

All statistical analyses were carried out on Graphpad Prism software. For *in vitro* studies, Student-t or one-way ANOVA test was used to compare two groups or more than two groups, respectively. For *in vivo* studies, log-rank test or Wilcoxon rank-sum test was used to compare symptom-free survival or tumor size, respectively. We considered differences significant when p < 0.05.

## Competing interests

The authors declare that they have no competing interests.

## Authors' contributions

MO, AN, and HO conceived and designed the experiments. MO, TO, AK, and KT performed the experiments. MO and HO analyzed the data. MO, AN, and CHJ contributed to securing reagents/materials/analysis tools. MO and HO wrote the manuscript. All authors read and approved the final manuscript.

## Supplementary Material

Additional file 1: Figure S1 Efficacy of CAR-T-cells in mice. Mice received i.c. inoculation of 5×104 U87-EGFRvIII cells on day -7, and subsequently received i.v. infusion of 2×106 CAR-T-cells with/without miR or mock-transduced T-cells on day 0. All mice received i.p. administration of TMZ (0.33 mg/mouse/day) on days 0–4. Colored images represent photon flux signals from U87EGFRvIII-Luc tumors. **Figure S2.** CAR-T-cells did not exert therapeutic effects without TMZ. Mice received i.c. inoculation of 1×105 U87-EGFRvIII-Luc cells on day -7, and subsequently received 2×106 T-cells on day 0. **(A)** Longitudinal measurements of tumor-derived mean photon flux ± SD. **(B)** Kaplan-Meier plots for survival of the mice treated with mock-transduced T-cells or CAR-T cells (with or without co-transduction of miR-17-92) (n = 5/group). **Figure S3.** Efficient homing and persistence of CAR-T-cells in U87-EGFRvIII tumors. Mice treated with TMZ and CAR-T-cells were sacrificed on day 14 following the tumor re-challenge per data in Figure 5. **(A and B)** A low (**A**; x4) or high (**B**; x20) magnification view of a section stained with hematoxylin and eosin. **(C and D)** CAR-T-cell infiltration in the i.c. tumor detected by immuno-fluorescence imaging with **(D)** or without **(C)** biotin-conjugated anti-F(ab’)2 mAb and streptavidin-PE. The counter staining with DAPI (blue) indicates cells in the tissue. **Figure S4.** The number of spleen cells in mice after CAR-T-cells treatment. Treated mice were sacrificed on Day 21 following the tumor re-challenge per data in Figure 5. The number of splenocytes were calculated and stained with anti-human CD8, CD4 and anti-mouse F(ab’)2 antibody for detection of CAR-expressing T-cells. N = 3/group. **Figure S5.** CAR-T-cell selection with magnetic beads for *in vitro* study. Three days after transduction of CAR, T-cells were selected using biotin-anti-mouse F(ab’)2 antibody in conjunction with streptavidin-ferromagnetic beads. The selected cells were stained with biotin-anti-mouse F(ab’)2 antibody and streptavidin-PE. Click here for file
