# Supplementary material for: Expression of miR-17-92 enhances anti-tumor activity of T-cells transduced with the anti-EGFRvIII chimeric antigen receptor in mice bearing human GBM xenografts
Source: J Immunother Cancer. 2013 Dec 16;1:21. doi: 10.1186/2051-1426-1-21 (PMC4019893; doi:10.1186/2051-1426-1-21)

Supplementary Figure Legends

Supplementary Figure S1. Efficacy of CAR-T-cells in mice. NSG mice received i.c. inoculation of $5x10^4$ U87-EGFRvIII cells on day -7, and subsequently received a single i.v. infusion of $2x10^6$ T cells transduced with pELNS-3C10-CAR alone or both pELNS-3C10-CAR and FG12-EF1a-miR-17/92, or mock vector on day 0. All mice received i.p. administration of TMZ (0.33mg/mouse/day) on days 0-4. Colored images represent photon flux signals from U87EGFRvIII-Luc tumor-derived luciferase activity.

Supplementary Figure S2. CAR-T-cells did not exert therapeutic effects without TMZ in mice bearing U87-EGFRvIII tumors. NSG mice received i.c. inoculation of $1x10^5$ U87-EGFRvIII-Luc cells on day -7, and subsequently received a single i.v. infusion of $2x10^6$ T-cells transduced with pELNS-3C10-CAR alone or both pELNS-3C10-CAR and FG12-EF1a-miR-17/92, or mock vector on day 0. (A) Longitudinal measurements of tumor-derived mean photon flux ± SD. The background luminescence level was defined based on the levels observed in non-tumor-bearing mice imaged in parallel with the tumor-bearing mice. (B) Kaplan-Meier plots for survival of the mice treated with mock-transduced T-cells or CAR-T cells (with or without co-transduction of miR-17-92) (n=5/group).

Supplementary Figure S3. Efficient homing and persistence of CAR-T-cells in U87-EGFRvIII tumors in the brain. Mice treated with TMZ and CAR-T-cells were sacrificed on day 14 following the tumor re-challenge per data in Figure 5. (A and B) A low (A; x4) or high (B; x20) magnification view of a section stained with hematoxylin and eosin (H&E). (C and D) CAR-T-cell infiltration in the i.c. U87-EGFRvIII tumor detected by immuno-fluorescence imaging with (D) or without (C) biotin-conjugated anti-F(ab')2 mAb and streptavidin-PE. The counter staining with DAPI (blue) indicates cells in the tissue.

Supplementary Figure S4. The number of spleen cells in mice after CAR-T-cells treatment. Mice treated with TMZ and CAR-T-cells were sacrificed on Day 21 following the tumor re-challenge (Day 70 since i.v. infusion of CAR-T-cells) per data in Figure 5, and spleens were harvested. The number of splenocytes were calculated and stained with anti-human CD8, CD4 and anti-mouse F(ab')2 antibody for detection of CAR-expressing T-cells. N=3/group. Bars and error bars indicate the median and standard deviations, respectively. There were no statistically significant differences between CAR-T-cells with (■) and without (□) miR.

Supplementary Figure S5. CAR-T cell selection with magnetic beads for *in vitro* study. Three days after transduction of pELNS-3C10-CAR, CAR-expressing cells were selected using biotin-anti-mouse F(ab')2 antibody in conjunction with streptavidin-ferromagnetic beads. The selected cells were stained with biotin-anti-mouse F(ab')2 antibody and streptavidin-PE, and analyzed by flow-cytometer.

Supplementary Figure S1

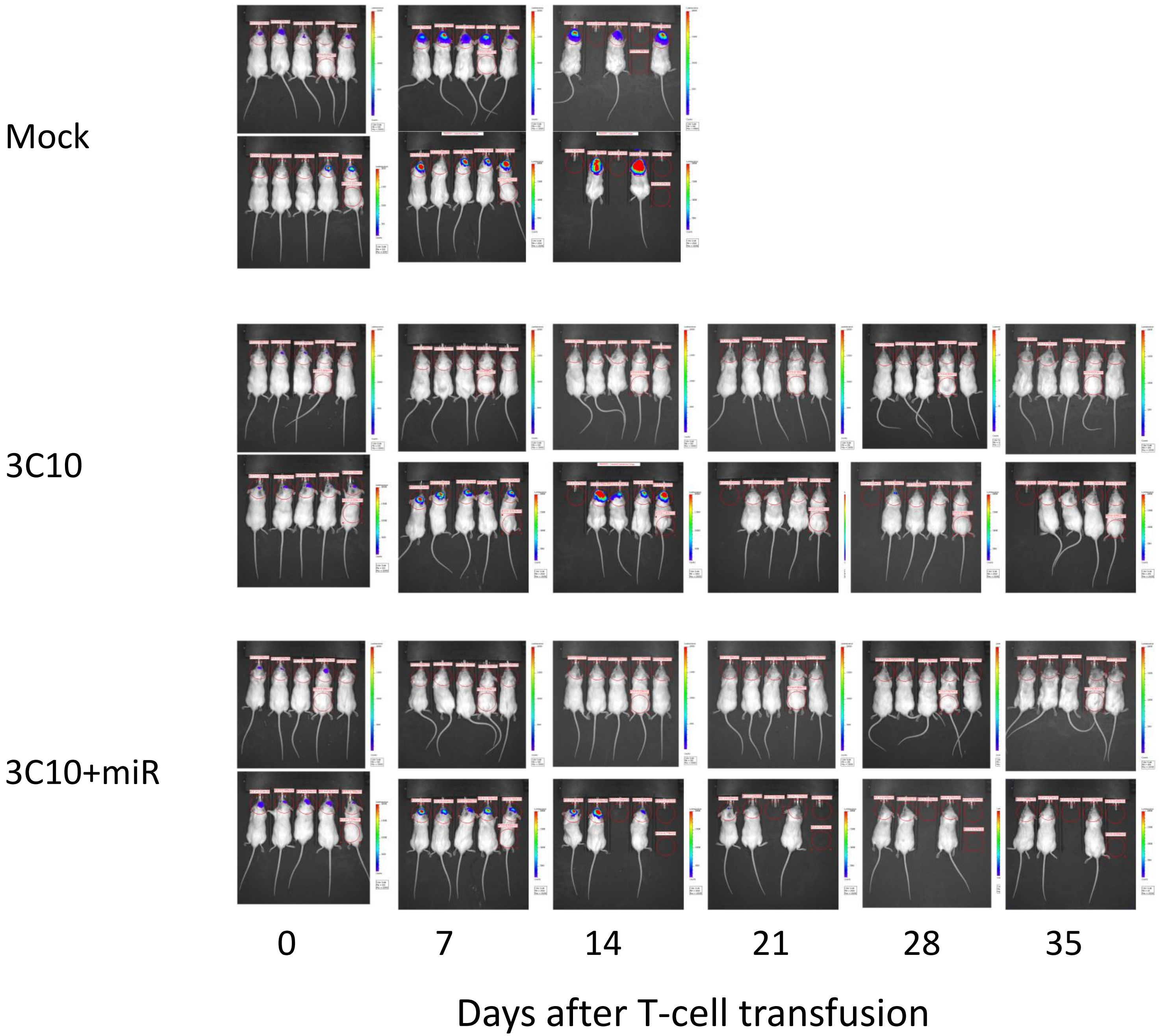

Supplementary Figure S2

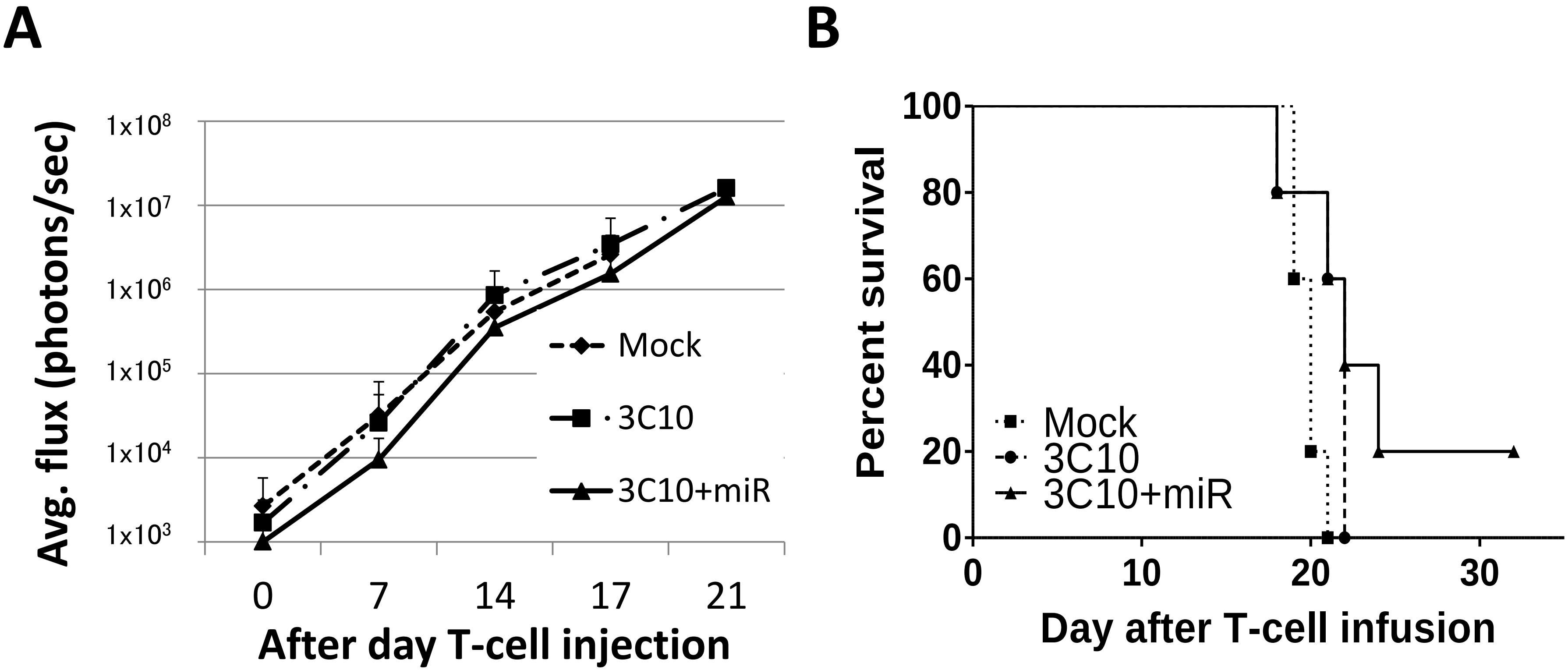

Supplementary Figure S3

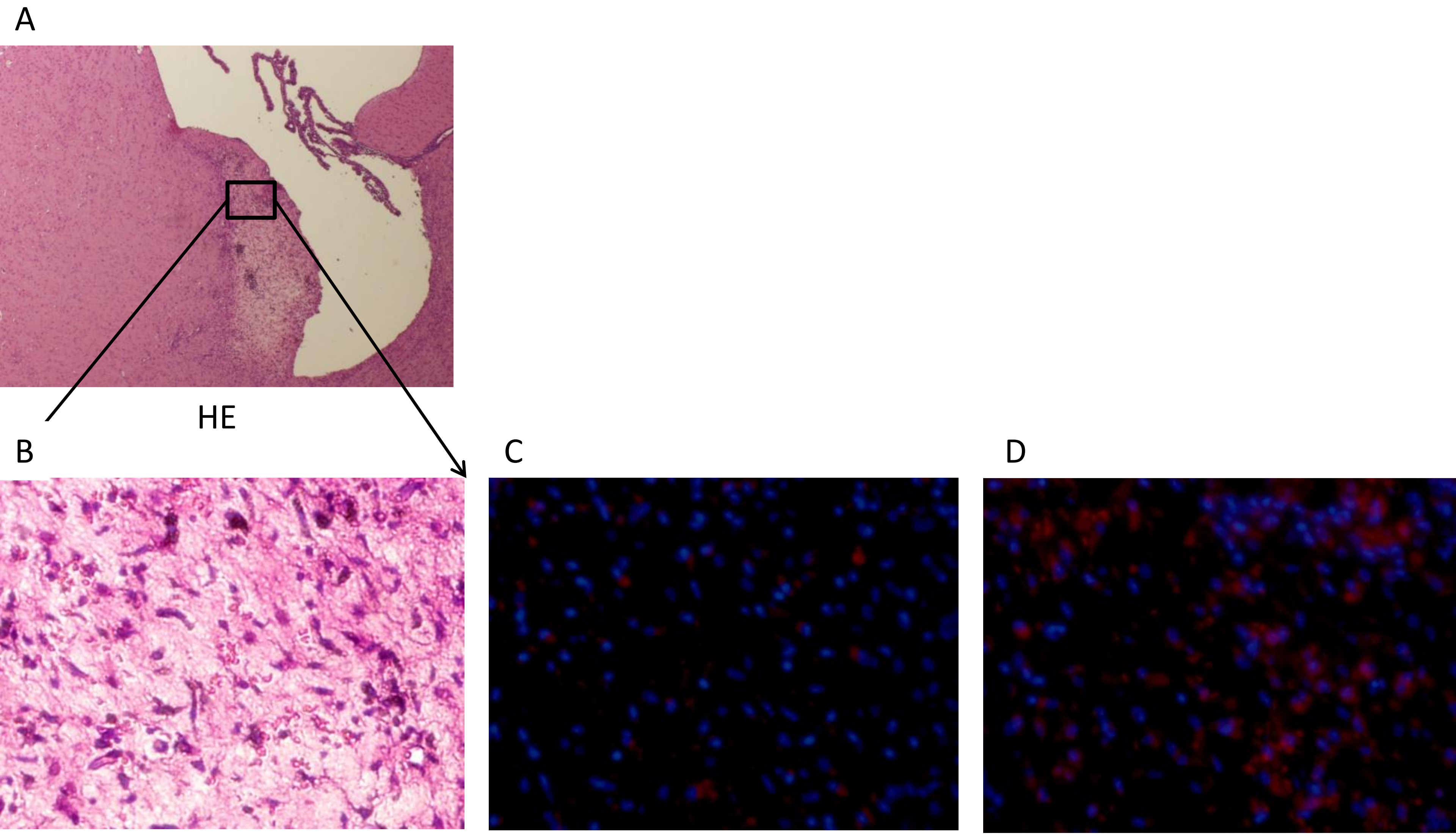

HE

Streptavidin-PE

Anti-mouse F(ab')2
+Streptavidin-PE

Supplementary Figure S4

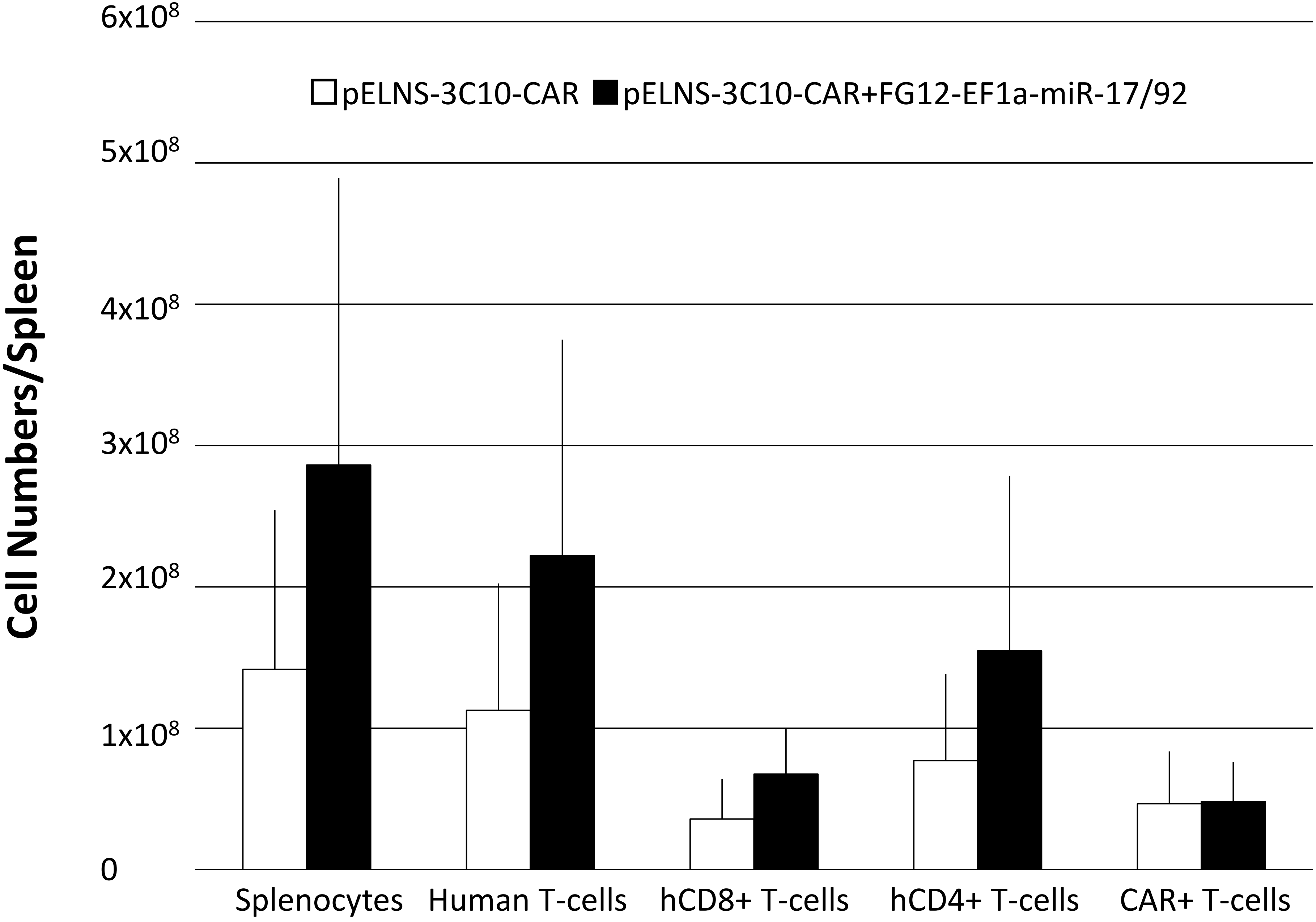

Supplementary Figure S5

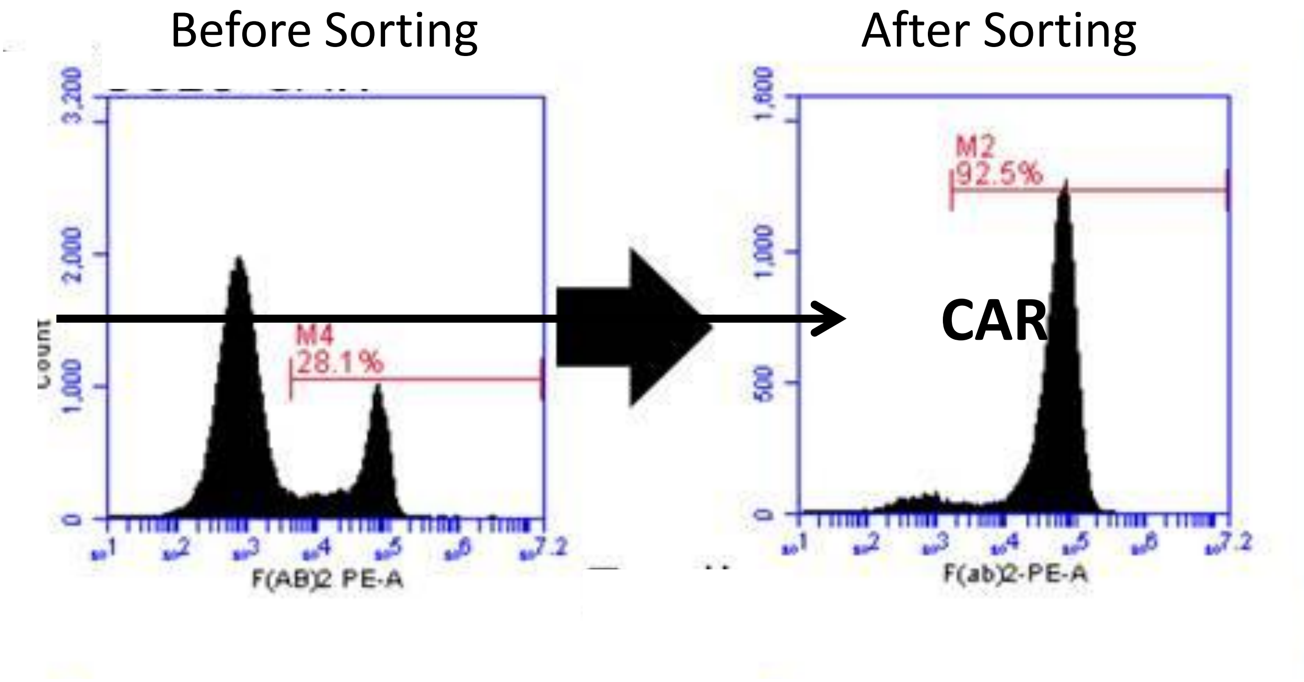

Supplement: Additional file 1: Figure S1 — Efficacy of CAR-T-cells in mice. Mice received i.c. inoculation of 5×10^4^ U87-EGFRvIII cells on day -7, and subsequently received i.v. infusion of 2×10^6^ CAR-T-cells with/without miR or mock-transduced T-cells on day 0. All mice received i.p. administration of TMZ (0.33 mg/mouse/day) on days 0–4. Colored images represent photon flux signals from U87EGFRvIII-Luc tumors. Figure S2. CAR-T-cells did not exert therapeutic effects without TMZ. Mice received i.c. inoculation of 1×10^5^ U87-EGFRvIII-Luc cells on day -7, and subsequently received 2×10^6^ T-cells on day 0. (A) Longitudinal measurements of tumor-derived mean photon flux ± SD. (B) Kaplan-Meier plots for survival of the mice treated with mock-transduced T-cells or CAR-T cells (with or without co-transduction of miR-17-92) (n = 5/group). Figure S3. Efficient homing and persistence of CAR-T-cells in U87-EGFRvIII tumors. Mice treated with TMZ and CAR-T-cells were sacrificed on day 14 following the tumor re-challenge per data in Figure 5. (A and B) A low (A; x4) or high (B; x20) magnification view of a section stained with hematoxylin and eosin. (C and D) CAR-T-cell infiltration in the i.c. tumor detected by immuno-fluorescence imaging with (D) or without (C) biotin-conjugated anti-F(ab’)2 mAb and streptavidin-PE. The counter staining with DAPI (blue) indicates cells in the tissue. Figure S4. The number of spleen cells in mice after CAR-T-cells treatment. Treated mice were sacrificed on Day 21 following the tumor re-challenge per data in Figure 5. The number of splenocytes were calculated and stained with anti-human CD8, CD4 and anti-mouse F(ab’)2 antibody for detection of CAR-expressing T-cells. N = 3/group. Figure S5. CAR-T-cell selection with magnetic beads for in vitro study. Three days after transduction of CAR, T-cells were selected using biotin-anti-mouse F(ab’)2 antibody in conjunction with streptavidin-ferromagnetic beads. The selected cells were stained with biotin-anti-mouse F(ab’)2 antibody and streptavidin-PE [file 2051-1426-1-21-S1.pdf]
